# Comprehensive analysis of pseudogenes in prokaryotes: widespread gene decay and failure of putative horizontally transferred genes

**DOI:** 10.1186/gb-2004-5-9-r64

**Published:** 2004-08-26

**Authors:** Yang Liu, Paul M Harrison, Victor Kunin, Mark Gerstein

**Affiliations:** 1 Department of Molecular Biophysics and Biochemistry, Yale University, PO Box 208114, New Haven, CT 06520-8114, USA; 2 Computational Genomics Group, The European Bioinformatics Institute, EMBL Cambridge Outstation, Cambridge CB10 1SD, UK; 3 Current address: Department of Biomedical Informatics, Columbia University, 622 W 168th street, New York, NY 10032, USA

## Abstract

**Background:**

Pseudogenes often manifest themselves as disabled copies of known genes. In prokaryotes, it was generally believed (with a few well-known exceptions) that they were rare.

**Results:**

We have carried out a comprehensive analysis of the occurrence of pseudogenes in a diverse selection of 64 prokaryote genomes. Overall, we find a total of around 7,000 candidate pseudogenes. Moreover, in all the genomes surveyed, pseudogenes occur in at least 1 to 5% of all gene-like sequences, with some genomes having considerably higher occurrence. Although many large populations of pseudogenes arise from large, diverse protein families (for example, the ABC transporters), notable numbers of pseudogenes are associated with specific families that do not occur that widely. These include the cytochrome P450 and PPE families (PF00067 and PF00823) and others that have a direct role in DNA transposition.

**Conclusions:**

We find suggestive evidence that a large fraction of prokaryote pseudogenes arose from failed horizontal transfer events. In particular, we find that pseudogenes are more than twice as likely as genes to have anomalous codon usage associated with horizontal transfer. Moreover, we found a significant difference in the number of horizontally transferred pseudogenes in pathogenic and non-pathogenic strains of *Escherichia coli*.

## Abstract 2

A comprehensive analysis of the occurrence of pseudogenes in a diverse selection of 64 prokaryote genomes identified around 7,000 candidate pseudogenes. A large fraction of prokaryote pseudogenes seems to have arisen from failed horizontal-transfer events.

## Background

Genes that have recently fallen out of use for an organism are often detectable in the genome as pseudogenes - disabled copies of genes characterizable by disruptions of their reading frames due to frameshifts and premature stop codons [1-3]. Surveys of the pseudogene populations of eukaryotes (budding yeast, nematode worm, fruit fly and human) have recently been completed [4-10]. These pseudogene analyses have yielded insights into eukaryotic proteome evolution, showing that duplicated pseudogene formation tends to occur in younger, more lineage-specific, protein families, and is in many cases linked to the generation of functional diversity [3]. However, pseudogene formation in most prokaryotes has not been analyzed as a matter of course, and has, historically, been assumed to be minimal [11]. Some recent substantial populations of pseudogenes have been discovered in pathogenic bacteria, most notably in the leprosy bacillus *Mycobacterium leprae*, where around 1,100 pseudogenes (compared to around 1,600 genes) were found, with pseudogene formation providing a 'fossil record' of recent wholesale loss of pathways involved in lipid metabolism and anaerobic respiration [12].

Here we want to address the question of whether these large populations are exceptional, or whether there are substantial populations of pseudogenes in other prokaryotic genomes. If so, from a holistic 'polygenomic' perspective, what sorts of proteins tend to form prokaryotic pseudogenes? And are there any themes in common with the occurrence of pseudogenes in eukaryotes?

To address these broad questions, we have adapted a pipeline developed for eukaryotic pseudogene identification to 64 prokaryotic genomes [4]. The species analyzed include archaea, pathogenic bacteria and non-pathogenic bacteria, and many of the pathogenic bacteria are also important organisms in current biodefense research. We have found nearly 7,000 pseudogenes, with notable numbers of pseudogenes for specific families linked to DNA transposition and also that have some role in environmental responses. Our results, which we have derived consistently across all the genomes, are available from our prokaryote pseudogene information website [13].

## Results and discussion

### Pseudogenes are pervasive in prokaryotes

**Figure 1:**
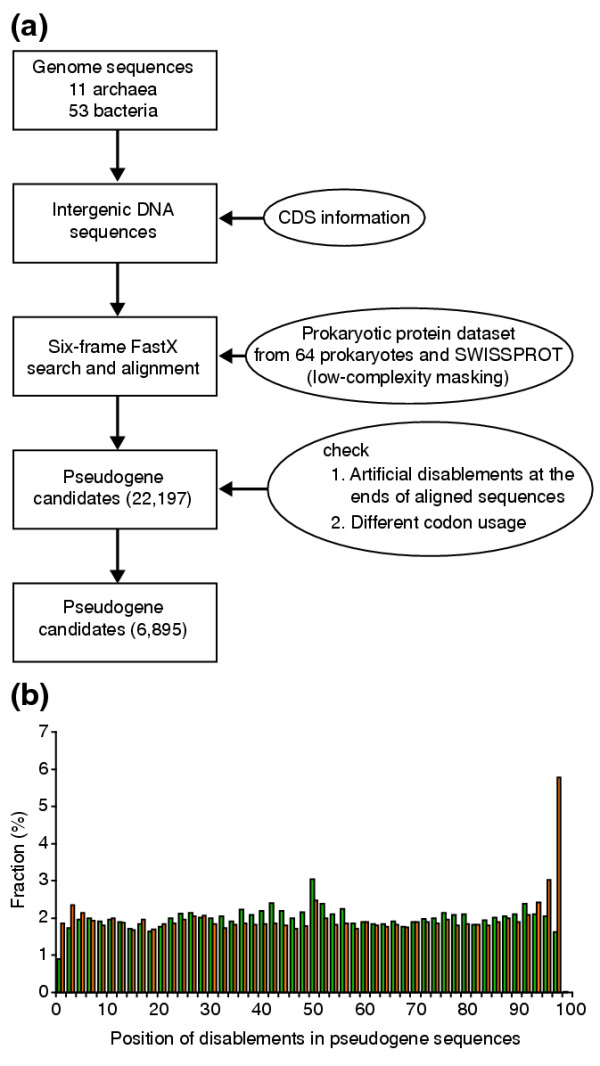
Pseudogenes in prokaryotes. **(a) **Procedure for assigning pseudogenes. The flow chart shows the steps in identifying pseudogenes in 64 prokaryote genomes. The steps include: separate intergenic regions from coding sequence (hypothetical ORFs were excluded); six-frame FastX search on intergenic regions for pseudogene candidates; quality control to reduce false-positive results introduced by artificial disablement or by different codon usage. **(b) **The occurrence of relative disablement positions in pseudogenes, which were normalized on a 100-residue scale based on ratios of the distances from starting residues to disablements to the length of pseudogenes. The yellow bars indicate the distribution of disablement positions before the last quality-control step and the green bars show the distribution after minimizing false-positive pseudogenes.

To identify pseudogenes in prokaryotic genomes, we performed a conservative and comprehensive search, as outlined in Figure 1 and Materials and methods. We used a proteome set consisting of sequences from the 64 genomes and Swiss-Prot [14] with relatively high confidence in annotation (that is, excluding those annotated as hypothetical proteins). Intergenic regions in prokaryotic genomes were searched against the proteome set using FastX [15] for homology matches with disablements as pseudogene candidates. We then applied several checks to reduce false positives (see Materials and methods). Overall, we found 6,895 candidate pseudogenes.

**Figure 2:**
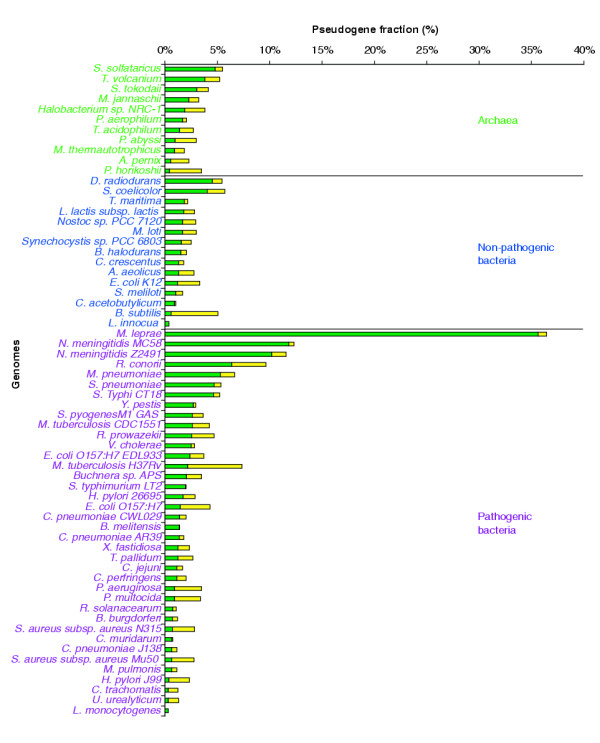
Fractions of pseudogenes in the 64 prokaryote genomes. The genomes are divided into three categories: archaea (green), non-pathogenic bacteria (blue) and pathogenic bacteria (purple). The yellow bars represent the fractions of pseudogenes that overlap with hypothetical ORFs, and the green bars represent those that do not overlap. Genomes in each category are sorted by the green bars.

Previously, the pseudogene fraction was defined as the ratio of the number of pseudogenes to the number of all gene-like sequences (genes plus pseudogenes) [16]. By this measure, we find that pseudogenes are pervasive in prokaryotes (Figure 2). Pseudogenes are detectable at a low 'background' level in most prokaryotes, ranging from 1 to 5% of the genome (Figure 2). Application of a more restrictive cutoff (E-value less than 0.001, instead of E-value less than 0.01) in FastX alignment results in slightly smaller percentage of pseudogenes (0.1% less on average) in all the genomes, and generates essentially the same results (data not shown). Our census is in general agreement with previous assessments of pseudogene content in the genomes of *M. leprae*, *Escherichia coli *and *Rickettsia prowazekii *[12,16-19]. In these previous studies, however, different criteria were used for pseudogene identification in different genomes, leading to inconsistencies in comparing results. This is avoided in our study by using a method applied uniformly across all genomes. All these assessments suggest that most prokaryotes have similar net genomic DNA deletion rates, resulting in similar low-level 'background' pseudogene fractions in their genomes.

To check for a correlation with microbial 'lifestyle', we classified the 64 species into three categories: archaea, pathogenic bacteria and non-pathogenic bacteria. The pseudogene fractions for these groupings were assessed. *M. leprae *has a very large pseudogene fraction (36.5%) and is clearly a unique outlier. When this genome is set aside, the three groups have similar pseudogene fractions (3.6%, 3.9% and 3.3%). Note that three other pathogenic species/strains have relatively large pseudogene fractions, including *Neisseria meningitidis *MC58 (12.4%), *N. meningitidis *Z2491 (11.6%) and *Rickettsia conorii *(9.7%). The higher pseudogene fractions of some pathogenic species have previously been suggested to be a result of a rapidly changing environmental niche, with loss of metabolic and respiratory pathways [12].

We found that about 2,300 of our 6,895 candidate pseudogenes overlap with more than 2,600 annotated hypothetical open reading frames (ORFs), whose fractions were indicated in Figure 2. The overlap could arise from erroneous gene annotations or sequencing errors [16]. In either case, the pseudogene annotation in prokaryotic genomes is evidently an important part of decontaminating gene annotation.

### Pseudogene families

**Figure 3:**
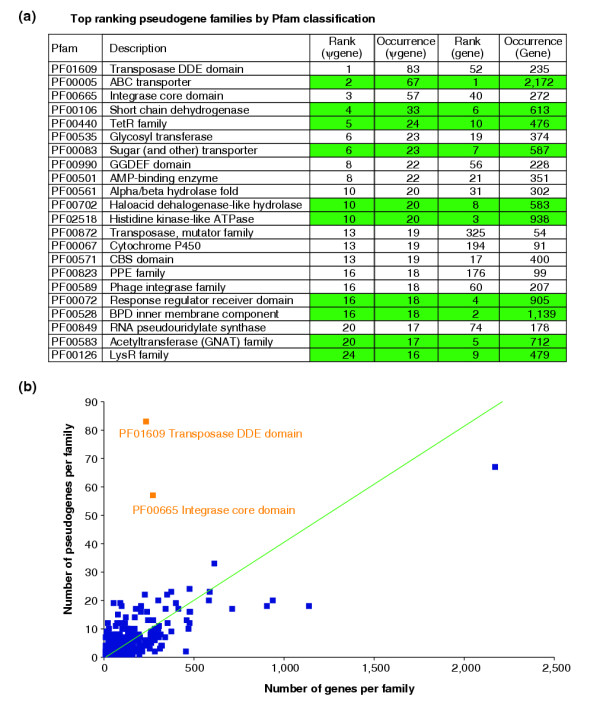
Gene-to-pseudogene ratios. **(a) **The top 20 pseudogene families and top 10 gene families based on Pfam classification. Ranking is based on the size of pseudogene families. The top 10 gene families are highlighted with the green background. **(b) **The number of genes plotted against the number of pseudogenes in a Pfam family. The line represents the overall ratio of the number of pseudogenes to the number of genes in the 64 genomes.

We used the Pfam classification [20] to analyze the families and functions of candidate pseudogenes. The 20 top-ranking domain families in terms of pseudogenes are shown in Figure 3a. Many large divergent gene families are among the top pseudogene families, including 9 of the top 10 gene families such as: the ABC transporter (PF00005), short-chain dehydrogenases/reductases (PF00106), sugar transporter (major facilitator superfamily) (PF00083), and histidine kinase-like ATPase (PF02518). As the largest family of proteins in prokaryotes, the ABC transporter functions to translocate a variety of compounds across biological membranes [21-23]. It consists of two ATP-binding domains (PF00005) [24,25] and two transmembrane domains (PF00664). These domains are present in large copy numbers across genomes (2,172 and 245 gene copies as well as 67 and 13 pseudogene copies respectively).

There are notable protein families that rank high in pseudogene number, but low in terms of gene number. They include the PPE family (PF00823) which is thought to be linked to antigenic variation in mycobacteria and is highly polymorphic [26]; the cytochromes P450 (PF00067), which are involved in processing diverse substrates; the GGDEF domain (PF00990), which is of unknown function and is associated with a wide diversity of other protein domains [27]; alpha/beta-hydrolase enzymes (PF00561), which have diverse catalytic functions; and pseudo-U-synthase-2 enzymes (PF00849), which help synthesize pseudouridine from uracil. Note that the first two families in this list have sequence diversity that has some link to environmental response.

Figure 3b shows the relationship between the number of pseudogenes and genes for Pfam families. One might expect this relationship to be linear, with bigger families having more pseudogenes, but Figure 3b shows this is not the case. Two large families that have a relatively high ratio of pseudogenes to genes are the transposase DDE domain (PF01609) and integrase core domain (PF00665). Transposase facilitates DNA transposition and horizontal gene transfer and its DDE domain may be responsible for DNA cleavage at a specific site followed by a strand-transfer reaction [28]. Many transposons contain transposases for their transposition [29,30]. We found that two strains of *N. meningitidis *(MC58 and Z2491) carry 26 and 22 copies of transposase pseudogenes, respectively, and have only 11 and 5 copies of transposase genes. In the MC58 strain, transposase pseudogenes have been found in most of the 29 remnant insertion sequences [31]. This suggests that *N. meningitidis *strains probably undergo high selection pressure for transposases. The integrase core domain family (PF00665) is the catalytic domain of integrase, which mediates integration of a DNA copy of a viral/bacteriophage genome into the host genome [32]. It catalyzes the DNA strand-transfer reaction by ligating the 3' ends of the viral DNA to the 5' ends of the integration site [32]. The large number of transposase and integrase pseudogenes might result from harmful foreign genes being disabled in transposable elements. Several species contain many integrase pseudogenes, including *Streptococcus pneumoniae, M. leprae, M. tuberculosis*, and *E. coli *strain O157:H7. The large number of pseudogenes relative to genes for these two gene families may reflect an overall high selective pressure for them - that is, a gene family that is rapidly duplicating and evolving may generate many pseudogenes.

### Origins of pseudogenes

Retrotransposition and genomic DNA duplication generate pseudogenes in mammals and other eukaryotes [2,3]. In contrast, in prokaryotes, based on the experience annotating *E. coli *and *M. leprae *[12,16], pseudogenes are suggested to arise from three process: the disablement of detectable native duplications; the decay of native single-copy host genes; and failed horizontal transfers.

However, the complete extent of the processes forming prokaryotic pseudogenes is not yet well understood. We realize that there are many methods of defining horizontal transfer [33-36] and an active debate on the best way of doing this [37,38], so we applied two independent methods to predict horizontal gene transfer events. The first method (GC-content) is based on the GC content bias at particular codon positions of recently acquired genes [33,39]. The second method (GeneTrace) is based on the analysis of phylogenetic distribution of protein families on species tree [40]. In the GC-content method, the number of pseudogenes resulting from horizontal transfer in each genome was estimated by applying the same criteria to them as had been previously used to identify horizontally transferred genes. Overall, we found that the ratio (19.9%) of pseudogenes from potential horizontal transfer to those derived from the host is significantly higher than the ratio of genes in the host (8.6%). We dubbed the ratio of these two quantities the 'failed horizontal transfer index', and observed that it implies that pseudogenes are 2.3 times more likely to arise from horizontal transfer than host genes are (Table 1).

To confirm our findings based on a method relying on GC content bias we applied the GeneTrace method (see Materials and methods). We analyzed a subset of pseudogenes and found that 18% result from failed horizontal transfer events, consistent with the previous method. Note that GeneTrace and the GC-content method are very different in the criteria they use to assess horizontal transfer and thus make for good independent verification of each other.

In summary, we report here for the first time an estimate of how often horizontal transfer in prokaryotes introduces genes that are redundant, useless or even detrimental. Firstly, ORFs from dangerous genetic elements are under strong selection pressure to be deleted from the host's genome [11]. Secondly, horizontally transferred genes have a higher chance than non-transferred genes of becoming pseudogenes in most prokaryotes, which may be a result of deactivation/disablement of non-beneficial transferred genes.

By examining closely related strains of the same species, we found that most close strains have a similar value for the failed horizontal transfer index. In particular, *M. tuberculosis *(strains H37Rv and CDC1551), *N. meningitidis *(strains Z1491 and MC8), and *Helicobacter pylori *(strains 26695 and J99) share similar index values within species. However, *E. coli *has different index values in the three strains studied. The free-living *E. coli *K12 strain has an index value of 4.6, comparable to values calculated from previous results [16], while the two pathogenic *E. coli *strains O157:H7 and O157:H7 EDL933 have much lower values (1.8 and 0.8). This can be readily explained in two ways: the intracellular pathogenic *E. coli *strains could have moved into a different environment that results in lower exposure to incoming DNA and thus to a lower rate of horizontal gene transfer [41]; or these strains could have an increased rate of gene loss or pseudogene formation of their host genes.

### A polygenomic power-law-like trend in pseudogene disablement

**Figure 4:**
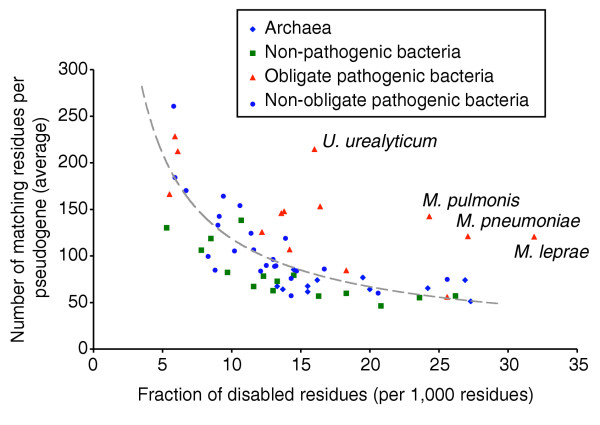
The fraction of disabled residues (per 1,000 residues) versus the number of average matching residues to the closest homologs per pseudogene in the 64 species categorized into four groups: archaea (blue diamonds), non-pathogenic bacteria (green squares), obligate pathogenic bacteria (purple circles) and non-obligate pathogenic bacteria (red triangles).

To characterize the overall rate of decay of pseudogene populations, we plotted the fraction of disablements versus the average number of matching residues (to their closest homologs) per pseudogene for each species. This measure shows how the overall level of decay of a pseudogene population relates to age (which corresponds to the degree of overall match to the closest homologs). There is a general power-law-like behavior governing this measure, with recent pseudogenes having few disablements and divergent pseudogenes having many (Figure 4). Archaea and most non-pathogenic bacteria cluster together at higher rates of disablement (between 10 and 28 per 1,000 residues) and less significant matches, indicating comparatively greater retention of ancient gene remnants in those species and fewer young pseudogenes. On the other hand, obligate pathogenic bacteria tend to have younger pools of pseudogenes, even though they exhibit high disablement rates. Interestingly, four species of obligate bacterial pathogens clearly stand out from the general tendency: these are *M. leprae *and three closely related mycoplasma species: *Mycoplasma pneumoniae*, *Mycoplasma pulmonis *and *Ureaplasma urealyticum*. Pseudogenes in these four pathogenic bacteria carry several times more disablements, suggesting that these bacteria have an accelerated disabling mutation rate. It is known that *M. leprae *has lost the *dnaQ*-mediated proofreading activities of DNA polymerase III [12,42], which could contribute to a higher mutation rate. The higher mutation rates in these species might suggest that these pathogens are under adaptation to their new environment, or have specific genome regions that are hypermutable.

It is important to note here that the current sequence databases are derived from an uneven sampling of genomes. Therefore, genomes of organisms with more sequenced relatives may appear to have, on average, a seemingly younger population of pseudogenes, while others may appear to have older and fewer identifiable pseudogenes. Using data from 64 genomes, our results indicate an overall trend for pseudogenes observed in most of the genomes studied. However, these results have to be viewed as preliminary until more genome data is available.

## Conclusions

We have shown that pseudogenes in prokaryotes are not uncommon, occupying 1-5% of all gene-like sequences. We find that specific gene families with clear links to DNA transposition and environmental responses have higher pseudogene/gene ratios.

The pseudogene data has many implications for the study of genome reduction and expansion [43,44]. A significant proportion of the pseudogenes arose from putative failed horizontal transfer - at more than two times the rate for genes. Obligate pathogenic bacteria have high rates of disablement in younger pseudogene populations, consistent with recent accelerated genome reduction [44], while, in contrast, archaea and non-pathogenic bacteria have relatively older pseudogene populations, but similar rates of disablement.

In terms of methodological implications, it is evidently necessary to include prokaryote pseudogenes as part of systematic annotation pipelines in the future. In addition, it was also shown to be helpful to identify potential short ORFs [45]. Furthermore, our survey shows that trends can be observed 'polygenomically' for prokaryotes, where they are not obvious or significant in individual genomes.

## Materials and methods

### Database releases used

We used the following datasets in our prokaryotic pseudogene analysis: Swiss-Prot (release 40.19 and updated to 27 May, 2002) [14] containing 43,094 prokaryotic protein sequences; nucleotide sequences from 64 prokaryotic genomes from EMBL database release 70 on March-2002 [46], including 11 genomes from archaea and 53 from bacteria as listed in Figure 1; Pfam release 7.3 of May 2002, containing 3,849 families and 498,152 protein domains in the alignments [20].

### Pseudogene identification pipeline

Figure 1a shows the basic procedure for identifying prokaryotic pseudogenes. The general schema was adapted from pipelines for pseudogene analysis in eukaryotes [4]. We generated a prokaryotic proteome set by collecting all the prokaryotic protein sequences in the Swiss-Prot database and those annotated in the 64 prokaryotic genomes. To be conservative, we did not include hypothetical or putative proteins, a large proportion of which might be overannotated [47,48]. All the protein sequences were masked by SEG using the default low-complexity filter parameters (122.22.5) [49]. To maximize the efficiency of the pseudogene search, we only considered the intergenic DNA regions in the 64 prokaryote genomes (including the regions encoding hypothetical proteins) as query sequences, and searched their forward and reverse complement sequences against the proteome set using FastX [15]. Significant homology matches (E-value less than 0.01) that contained more than one disablement (either a frameshift caused by insertion or deletion of nucleotides or a premature stop codon) were considered as potential pseudogenes. If an intergenic region had multiple matches, these matches were sorted by E-value (increasing) and then by the number of matching residues (decreasing), if they have the same E-value. The match with the most significant E-value and the maximum matching residues was selected and redundant matches were removed.

To ensure that spurious disablements were not introduced at ends of sequences as an alignment artifact, we excluded homology matches whose disablements occurred only within a 'cutoff region' at either end. We used 16 residues for the cutoff region for short sequences (160 amino acids or fewer) - a parameter that has been applied previously [6]. For longer sequences (more than 160 amino acids), 10% of the sequence length was applied as the cutoff region as FastX tends to include more residues at the ends of alignments.

We also assessed the potential pseudogenes by examining the distribution of the disablements within pseudogene sequences. Given that mutations within pseudogenes are unconstrained, we would expect disablements on pseudogenes to be evenly distributed. Figure 1b shows the position of disablements within pseudogene fragments whose length is normalized to 100 residues. By removing those potential pseudogenes that only had disablements at their flanking regions at both ends, the distribution is almost evenly distributed. We used it as a 'control filter' to minimize false-positive pseudogenes. In the final pseudogene set, the length of pseudogenes ranges from 33 to 4,969 amino acids, with a median length of 130 amino acids, as compared with the proteome set, where the length ranges from 7 to 10,920 amino acids with a median length of 291 amino acids.

We considered non-standard codon usage in some bacteria, such as when TGA encodes tryptophan rather than a stop codon in mycoplasma species, including *Mycoplasma pneumoniae*, *M. pulmonis *and *U. urealyticum*. By manual examination of *E. coli *genes with translational frameshifts in the RECODE database [50], we found that those genes were included in coding sequences (CDS) and therefore were excluded from our pseudogene search.

Sequencing errors could also be a potential problem in the detection of pseudogenes. However, this effect is expected to be small, as comparison of independently sequenced isolates of the same *E. coli *strains indicated that only about 7% of candidate pseudogenes could be due to sequencing error [16]. To further consider the possibility of sequencing error, we examined the stop codons in the pseudogenes detected in the *S. pneumoniae *genome (frameshift positions are not considered as they are difficult to locate.). This genome and eight others found in the trace archive of the National Center for Biotechnology Information (NCBI) [51] and Ensembl [52] were all sequenced by TIGR. We selected *S. pneumoniae *as a case study as it is a relatively big genome available in the archive. By adapting a previous method [53], we examined the overall quality values (Q) for each nucleic acid of stop codons in the pseudogenes. Pseudogene sequences were aligned to the archived sequences (≥ 95% identity), and the quality values for nucleotides in stop codons were summed up. We chose 10^-2 ^as a cutoff of the error rate (err = 10^SUM(-0.1Q)^) for all nucleic acids. The stop codons with all three nucleic acids above the cutoff were validated. Out of 116 pseudogenes in this genome, 73 were found to contain 150 stop codons in total. Using the available data in the trace archive, we identified 54 pseudogenes with stop codons being aligned with the original sequences, and validated 47 of these (87%). In addition, a similar fraction of stop codons (101 out of 116) was confirmed.

### Family classification of genes and pseudogenes

All genes in the 64 genomes were assigned to Pfam families by cross-referencing of their Swiss-Prot ID. Pseudogenes were assigned to Pfam families through ID of their closest homologs. Only the homologs that cover more than 70% of the Pfam domain were selected. A pseudogene could be assigned to multiple Pfam families if it contains multiple domains.

### Estimation of horizontally transferred genes and pseudogenes

Here we used a method (GC-content) to estimate horizontal transferred genes on the basis of their base compositions [33,39]. We analyzed each of the 64 genomes individually, and atypical genes and pseudogenes were identified if the GC content at first and third codon positions was two or more standard deviations higher or lower than the mean values at those positions in genes.

To ensure that we had the codon positions accurately assigned for the GC-content method, we only analyzed codons for pseudogenes that aligned well with annotated protein sequences, specifically excluding the regions of the alignment around frameshifts. While it is true that the local alignment in some regions of a pseudogene may be ambiguous, causing some difference in the GC-content calculation in that region, the impact on the overall GC-content estimation is minimal, given how many positions we average over to calculate the failed transfer index score.

The results for the 64 genomes are shown in Table 1. The failed transferred index in the last column represents the ratio of the fraction of putative horizontally transferred pseudogenes to the fraction of horizontally transferred genes

,

similar to the measure previously used in *E. coli *[16]. This essentially gives a likelihood ratio for horizontal transfer for pseudogenes relative to that of genes.

Note that to minimize the effect of more divergent sequence alignments, for the horizontal-transfer calculations we only analyzed 1,748 'recent' pseudogenes, which have more than 50% sequence identity to their closest matches over an aligned subsequence of more than 100 residues.

We have investigated the statistical robustness of the failed transfer index using resampling approaches [54]. For each of the 64 genomes, we randomly picked 90% of its genes and calculated their GC content. Using the new GC content, we then identified the putative horizontally transferred genes and pseudogenes and calculated the failed transfer index. We applied the process 1,000 times, generating a distribution of 1,000 indexes, which has a mean value of 2.32 with standard deviation of 0.01.

We also applied an alternative method (GeneTrace) to estimate horizontally transferred pseudogenes [40]. In this method, potential horizontal transfer events are inferred within a protein family when it is present only in distantly related species and is absent from members of the same phylogenetic clade. We analyzed a subset of pseudogenes - 225 pseudogenes across 62 genomes - whose closest Swiss-Prot homologs share more than 70% sequence identity across at least 100 amino acids, and identified 41 of them (18%) as from failed horizontal transfer events.

## Figures and Tables

**Table 1:** Putative horizontally transferred genes and pseudogenes

Species	Gene		Pseudogene		Failed transfer index
			
	All	HT	All	HT	
**Archaea**					
*A. pernix*	615	45	4	2	6.8
*S. solfataricus*	2,235	231	48	6	1.2
*S. tokodaii*	1,797	185	35	19	5.3
*P. aerophilum*	1,855	171	10	3	3.3
*Halobacterium sp. NRC-1*	1,383	100	1	1	13.8
*M. thermautotrophicus*	1,350	122	5	5	11.1
*M. jannaschii*	1,280	106	15	8	6.4
*P. abyssi*	891	75	6	2	4.0
*P. horikoshii*	553	50	8	0	0.0
*T. acidophilum*	1,169	106	5	4	8.8
*T. volcanium*	1,061	100	16	6	4.0
					
**Non-pathogenic bacteria**					
*A. aeolicus*	1,244	107	3	0	0.0
*Synechocystis sp. PCC 6803*	2,696	237	5	1	2.3
*Nostoc sp. PCC 7120*	3,672	332	10	2	2.2
*S. coelicolor*	6,012	536	14	4	3.2
*B. halodurans*	3,279	299	11	3	3.0
*B. subtilis*	1223	102	44	3	0.8
*L. innocua*	2,924	263	1	1	11.1
*C. acetobutylicum*	3,129	295	5	1	2.1
*L. lactis subsp. lactis*	1,870	156	13	2	1.8
*C. vibrioides*	2,699	231	6	1	1.9
*M. loti*	5,235	476	14	3	2.4
*S. meliloti*	2,985	240	9	6	8.3
*E. coli K12*	2,897	230	63	23	4.6
*T. maritima*	1,445	137	8	0	0.0
*D. radiodurans*	1,964	134	9	1	1.6
					
**Pathogenic bacteria**					
*Buchnera sp. APS*	477	42	5	2	4.5
*U. urealyticum*	467	40	2	1	5.8
*M. pneumoniae*	610	55	30	19	7.0
*B. burgdorferi*	590	63	1	0	0.0
*M. pulmonis*	595	53	2	1	5.6
*C. trachomatis*	597	67	3	1	3.0
*C. muridarum*	815	81	2	0	0.0
*R. prowazekii*	504	49	7	1	1.5
*T. pallidum*	727	64	12	5	4.7
*C. pneumoniae J138*	839	74	1	0	0.0
*C. pneumoniae AR39*	831	70	5	1	2.4
*C. pneumoniae CWL029*	845	71	7	0	0.0
*R. conorii*	695	67	9	0	0.0
*M. leprae*	1,440	119	271	53	2.4
*C. jejuni*	1,291	108	2	0	0.0
*H. pylori J99*	856	70	5	1	2.4
*H. pylori 26695*	1,055	90	13	3	2.7
*S. pyogenes M1 GAS*	1,348	108	14	1	0.9
*S. pneumoniae*	1,632	114	54	2	0.5
*N. meningitidis Z2491*	1,432	112	26	4	2.0
*P. multocida*	1,035	96	7	2	3.1
*N. meningitidis MC58*	1,466	121	44	14	3.9
*X. fastidiosa*	1,550	152	15	1	0.7
*S. aureus subsp. aureus N315*	1,557	140	4	2	5.6
*S. aureus subsp. aureus Mu50*	1,563	138	4	2	5.7
*L. monocytogenes*	2,799	231	2	0	0.0
*C. perfringens*	1,943	165	2	0	0.0
*B. melitensis*	2,948	216	5	0	0.0
*R. solanacearum*	3,032	252	5	0	0.0
*V. cholerae*	2,846	216	24	5	2.7
*M. tuberculosis CDC1551*	2,837	262	49	7	1.5
*M. tuberculosis H37Rv*	1,446	130	38	4	1.2
*Y. pestis*	3,533	282	51	4	1.0
*S. typhi CT18*	3,986	338	147	18	1.4
*S. typhimurium LT2*	4,308	349	22	5	2.8
*E. coli O157:H7*	3,424	266	120	16	1.7
*E. coli O157:H7 EDL933*	4,322	353	73	5	0.8
*P. aeruginosa*	3,716	281	7	3	5.7
Total	123,420	10,571	1,458	290	2.3

All genes and pseudogenes and the fraction having atypical codon-position-specific GC contents in the 64 genomes studied. The failed horizontal transfer index was computed as described in Materials and methods.
